# Luminescent Fe_3_O_4_ Nanohybrid for Intra‐Cellular Imaging and Combinatorial Chemo–Photothermal Therapy in Cancer

**DOI:** 10.1002/cbic.202500886

**Published:** 2026-04-21

**Authors:** Bijaideep Dutta, Sonali Gupta, Sharanaya Purandare, Premlata Bind, Rudheer Bapat, Nand Kisore Prasad, Kanhu Charan Barick, P. A. Hassan

**Affiliations:** ^1^ Chemistry Division Bhabha Atomic Research Centre Mumbai India; ^2^ Homi Bhabha National Institute Mumbai India; ^3^ Department of Condensed Matter Physics and Materials Science Tata Institute of Fundamental Research Mumbai India; ^4^ Department of Metallurgical Engineering IIT BHU Varanasi India

**Keywords:** carbon dot, combination therapy, drug delivery, Fe_3_O_4_ nanoparticles, photothermal therapy

## Abstract

Highly stable water dispersible carbon dot decorated hybrid magnetic nanoparticles (FCDs) that combines magnetic Fe_3_O_4_ nanocrystals and fluorescent carbon dots (GUCDs) were successfully synthesized using a facile strategy. The resultant Fe_3_O_4_@GUCDs (FCDs) hybrid NPs not only validate excellent magnetic responsive properties (M_s_ = 33.5 emu g^−1^) from the magnetic (Fe_3_O_4_) core, but also exhibit intriguing photo luminescent properties, including excellent photo stability from the CDs produced from glucose–urea mixture. The FCDs can enter the intracellular region and illuminate breast cancer cells (MCF‐7), and showed a time and dose‐dependent localization kinetics. Meanwhile, the presence of hydrophilic surface functional groups in FCDs contributes to the excellent stability in aqueous solutions as well as loading of the anti‐cancer drug doxorubicin with a loading capacity of 22 mg g^−1^. More importantly, the FCDs can absorb NIR irradiation followed by their conversion to heat energy to achieve photothermal modality. Thus, such nanostructured hybrid NPs (FCDs) demonstrate great potential towards advanced single platform nano‐theranostics with combinatorial chemo–photothermal therapy.

## Graphical abstract

Schematic representation of luminescent nanohybrids for image‐guided chemo–photothermal therapy.

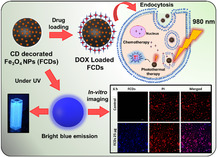

## 1 Introduction

The advent of nanotechnology has revolutionized the landscape of biomedical sciences, offering promising solutions for disease diagnosis, targeted drug delivery, and precision therapy [1, 2, 3, 4]. In particular, nanomaterials such as luminescent carbon dots (CDs) and magnetic iron oxide (Fe_3_O_4_) nanoparticles have garnered significant attention for their potential in biomedical applications [5, 6, 7, 8]. Carbon dots are well‐known for their tunable fluorescence, high photostability, low toxicity, and excellent biocompatibility, making them ideal candidates for use in bioimaging, biosensing, and drug‐tracking applications [9, 10, 11]. Conversely, Fe_3_O_4_ nanoparticles, recognized for their superparamagnetic behavior and high magnetic susceptibility, have been widely used in magnetic resonance imaging (MRI), drug delivery and magnetic hyperthermia therapy [12, 13, 14, 15, 16, 17, 18].

Magnetic nanoparticles (MNPs) and fluorescent materials together are highly valued as multimodal fluorescent–magnetic hybrid nanomaterials, primarily because their combined properties enable both diagnostic and therapeutic capabilities within a single nanoplatform. These systems allow simultaneous imaging, targeting and treatment, providing real‐time feedback on therapeutic progress and biodistribution. Traditionally, a range of organic dyes were employed as fluorescent probes in combination with magnetic nanoparticles for biological studies [19, 20, 21, 22]. However, owing to their short excited‐state lifetimes, small stokes shifts, narrow absorption bandwidths, pH sensitivity, and rapid photobleaching, conventional dyes suffer from poor stability and low performance in complex biological environments [23, 24, 25]. Semiconductor quantum dots (QDs) were introduced as an alternative due to their high quantum yields and broad excitation spectra, yet their inherent toxicity, limited biocompatibility, and environmental impact have significantly restricted their clinical translation [26, 27, 28].

Single‐mode nanoplatforms designed exclusively for fluorescence imaging, MRI or chemotherapy often face intrinsic performance limitations. For example, fluorescence imaging offers high sensitivity but suffers from limited tissue penetration, while magnetic or CT‐based systems provide deep tissue imaging but lack molecular specificity [29, 30]. In contrast, multimodal diagnostic and therapeutic systems (often termed theranostic nanoplatforms) integrate two or more imaging or therapeutic modalities into one construct, thereby compensating for individual shortcomings. Such systems provide complementary diagnostic information (e.g., anatomical via MRI and molecular via fluorescence), enable precise spatiotemporal tracking of drug delivery, and improve therapeutic outcomes by combining modalities such as chemo–photothermal or magneto–photothermal therapy [31, 32, 33]. Moreover, multimodal systems reduce systemic toxicity by enabling targeted delivery and controlled release, leading to enhanced therapeutic efficiency and reduced side‐effects. This integration is thus a crucial step toward precision nanomedicine and personalised therapy.

In recent years, numerous multimodal nanocomposites have been reported, each combining distinct functionalities for imaging and therapy. Typical examples include Au@Fe_3_O_4_, SiO_2_@Fe_3_O_4_, graphene oxide@Fe_3_O_4_, upconversion nanoparticle@Fe_3_O_4_, and carbon dot@Fe_3_O_4_ hybrids. For instance, Lipengolts et al. developed Fe_3_O_4_@Au core shell nanoparticles for bimodal CT/MRI imaging that exhibited excellent tumor accumulation, prolonged circulation, and minimal toxicity [34]. Similarly, Muzzi et al. demonstrated star‐shaped magnetic–plasmonic Au@Fe_3_O_4_ architectures that integrate plasmonic and magnetic properties for combined MR/CT/photoacoustic imaging and photothermal therapy [35]. Recent comprehensive reviews have emphasized that such multifunctional nanoparticles are redefining cancer theranostics by combining diagnostic imaging, targeted delivery and therapy within a single system [36, 37]. The construction strategies of these multimodal hybrids vary depending on the intended function and compatibility of the components. Common approaches include: Core–shell configuration, where a magnetic or metallic core (e.g., Fe_3_O_4_) is encapsulated by a fluorescent or plasmonic shell (e.g., Au, SiO_2_ or CDs) to enhance imaging and stability. These strategies not only optimise physicochemical and optical properties but also ensure biocompatibility, dispersibility, and controlled magnetic as well as fluorescent response. Despite this progress, the quest remains for simple, green, and reproducible synthetic routes that yield water‐dispersible, photostable, and non‐toxic multimodal nanohybrids suitable for in vivo translation. Hence, the integration of carbon dots with iron oxide nanoparticles merges the best of both materials; the luminescent properties of CDs enable high‐resolution fluorescence imaging, while the magnetic properties of Fe_3_O_4_ facilitate MRI contrast enhancement and magnetic targeting. This combination not only enhances diagnostic precision but also improves therapeutic efficiency through synergistic chemo–photothermal mechanisms.

In this study, we report the synthesis of a biocompatible, water‐dispersible, and luminescent carbon dot‐decorated magnetic iron oxide nanohybrid (Fe_3_O_4_@GUCDs; FCDs) for multimodal cancer theranostics. Nitrogen‐doped carbon dots (GUCDs), derived from glucose–urea precursors, exhibited strong blue fluorescence with excitation‐dependent emission (*λ*_ex_ = 280 nm, *λ*_em_ = 420 nm). Surface conjugation with Fe_3_O_4_ nanoparticles induced a hypsochromic shift (*λ*_em_ = 400 nm), attributed to interfacial charge transfer and modulation of surface states. The FCDs displayed uniform size distribution, high colloidal stability, and optimized surface charge, as confirmed by TEM, XPS, DLS, and zeta potential analyses. Under 980 nm laser irradiation, FCDs exhibited significant photothermal conversion efficiency, demonstrating their potential for near‐infrared (NIR)‐triggered hyperthermia. In vitro studies in MCF‐7 cells revealed enhanced chemo–photothermal cytotoxicity, while confocal microscopy confirmed efficient, time‐dependent intracellular uptake with strong fluorescence signals. The nanohybrids combine magnetic responsiveness, optical traceability, and photothermal functionality, enabling integrated diagnosis and therapy. Collectively, the study establishes FCDs as a robust nanotheranostic platform with excellent aqueous dispersibility, photostability, and low cytotoxicity, offering a versatile approach toward image‐guided, personalized cancer treatment.

## 2 Materials and Methods

### 2.1 Materials

Ferrous chloride tetrahydrate (FeCl_2_·4H_2_O, ≥99%), ferric chloride hexahydrate (FeCl_3_·6H_2_O, ACS reagent, 97%), glucose, and urea (AR grade) were purchased from Sigma Aldrich, USA. SDS (99%) and ammonia solution (25%) were bought from SRL Pvt. Ltd., India. Dulbecco’s Modified Eagle Medium (DMEM), fetal calf serum (FCS), MTT reagent (thiazolyl blue tetrazolium bromide), and dialysis membrane‐60 were procured from Himedia Laboratories Pvt. Ltd., India. 1, 10‐phenanthroline monohydrate were obtained from Alfa Aesar, Canada, and Merck, India, respectively. MCF‐7 (CVCL_0031) (Michigan cancer foundation) cells (human breast cancer) and A549 cells (CVCL_0023) (human lung cancer) were purchased from National Centre for Cell Science (NCCS), Pune, India. The acetate buffer (AB, pH 4.5) and phosphate‐buffered saline (PBS, pH 7.4) were prepared using standard protocols. All chemicals used were of AR grade unless otherwise specified. All the aqueous solutions were prepared using deionized water from a Millipore‐MilliQ system (resistivity ∼18 MΩ cm).

### 2.2 Synthesis of N‐Doped Carbon Dot (GUCDs)

The blue fluorescent carbon nanodots (GUCDs) were synthesized in a solvent‐free manner. 600 mg glucose (dextrose) and 400 mg were mixed in 6:4 ratio. After grinding nicely, white powdery mixture was transferred in glass vial. The vial was then placed in a paraffin oil bath for heating at 100–120°C. The mixture started to melt near 70°C, further increase in temperature turned mixture brownish from white between 100–120°C with continuous stirring which hints the formation of carbon dot. Reaction mixture was left in oil for rest 10–15 min, considering complete conversion of glucose and urea into carbon dot (GUCDs). The as‐prepared GUCDs were then purified using dialysis membrane of 1kDa for 24 h against nanopure water. The purified GUCDs were lyophilized and stored for further use.

### 2.3 Synthesis of Bare Iron Oxide Nanoparticles (Fe_3_O_4_)

Magnetic iron oxide was produced in combination of ferrous chloride (FeCl_2_·4H_2_O) and ferric chloride (FeCl_3_·6H_2_O) by co‐precipitation process as described earlier [38]. Briefly, 0.994 g of FeCl_2_·4H_2_O and 2.703 g of FeCl_3_·6H_2_O (molar ratio of Fe^2+^/Fe^3+^ = 1: 2) were dissolved in 40 mL of Milli Q water in a round‐bottom flask, and the temperature was slowly increased to 70^0^C under a nitrogen atmosphere with constant stirring. The temperature was maintained at 70^0^C for 30 min, and then 15 mL of 25% ammonia solution was added immediately to the reaction mixture and kept for another 60 min at 70^0^C. After that, the reaction mixture was allowed to cool down and the obtained black‐colored precipitates were thoroughly rinsed three times with Milli Q water and separated from the supernatant using a permanent magnet (field strength 2.5 kOe). The washed Fe_3_O_4_ particles were kept for drying. These dried black magnetic Fe_3_O_4_ particles were reserved for further use.

### 2.4 Synthesis of Carbon Dot (GUCDs) Decorated Iron Oxide Nanoparticles (FCDs)

Carbon dot decorated iron oxide nanoparticles (FCDs) were synthesized using a combinational approach. The precursor of GUCDs was finely grinded and transferred to a glass vial kept in oil bath. As the mixture started to melt at 70^0^C, Fe_3_O_4_ (60 mg) was added to it. The temperature was then raised to 120^0^C which led to the change of brown melted concoction to black after 1 h. The mixture was allowed to cool. These black crude product (FCDs) was first washed with water–ethanol mixture and separated using permanent magnet to remove all the unreacted precursor molecules. The FCDs were then made lyophilized for all future investigations.

### 2.5 Characterization

The structural/microstructural studies and surface functionalization were performed by X‐ray diffraction (XRD, Rigaku X‐ray diffractometer), transmission electron microscopy (FEI Tecnai 20, having LaB_6_ emitter, operated at 200 KV), and Fourier transform infrared (FTIR) spectroscopy (Bomem MB series). The average hydrodynamic diameter and zeta‐potential measurements were performed using a Litesizer (Anton Paar). The absorption spectra were recorded using a UV−visible spectrophotometer (JASCO V‐670 UV−vis spectrophotometer). The concentration of Fe in an aqueous suspension of FCDs was measured using an atomic absorption spectrometer (AAS, GBC Savant). For AAS analysis, the FCDs NPs were completely digested in aqua regia to ensure that their elemental components remain in solution.

### 2.6 Drug Loading and Release Study

Doxorubicin (DOX) drug loading was performed by taking 1:10 drug‐to‐particle ratio. For that purpose, 5 mg FCDs NPs (2.5 ml) were added into a DOX (0.5 ml of 1 mg/ml) solution and kept under vortexing at 37°C for 2 h in dark. Then, the DOX‐loaded particles (DOX@FCDs) were magnetically separated from the unloaded drug molecules. For determination of loading efficiency (%) of DOX, the absorbance of supernatant solution (obtained after magnetic separation of loaded drug) was recorded at 480 nm with respect to that of pure DOX. The loading efficiency was obtained as follows:

$$\text{Loading efficiency }\left(\text{\%}\right)=\frac{\left(A_{\mathrm{DOX}}‐A_{S}\right)}{\left(A_{\mathrm{DOX}}\right)}\times 100\;$$

where, *A*_DOX_ is absorbance of standard DOX, *A*_S_ is absorbance of supernatant solution (amount of pure DOX was kept same as that used during loading experiment).

The in vitro release test of DOX from the FCDs NPs was evaluated by the dialysis method under reservoir (r) − sink (s) conditions at 37°C. The DOX@FCDs were redispersed in 5 ml of respective release medium (AB pH 5.5 or PBS pH 7.4) and then transferred into a dialysis membrane. The drug release experiment was performed against 100 ml of AB/ PBS under continuous stirring at 37°C. 1 ml of the external solution (from sink) was removed at different intervals of time and replenished with respective fresh buffer to retain the sink conditions [39]. The percentage of drug released was obtained by measuring the fluorescence intensity at 590 nm (*λ*_ex_: 485 nm) using a plate reader with respect to the standard plot (measured under the same conditions). The standard deviation (SD) obtained from triplicate measurements is shown in the respective figures.

### 2.7 Photothermal Heating Study

Photothermal studies were performed by irradiating NIR light (980 nm) on 1 mL of aqueous suspension of FCDs (250 µg/ml 125 µg/ml Fe content in FCDs) NPs in a falcon tube at external laser powers of 0.9 W. The laser light was irradiated on sample suspension at 15 mm distance through a Fiber connector (Fiber core: 400 μm, NA: 0.22) attached to a CW 980 nm diode laser (SDL‐980‐LM‐2000T, Shanghai Dream Lasers Technology Co. Ltd., Shanghai). The change in temperature of solution was measured using a fiber optic signal conditioner (accuracy = ±0.2°C) at every 30 s (FOTEMP1‐H, Micronor Inc., US). In order to investigate the photostability, an aqueous suspension of FCDs NPs was irradiated with a 980 nm NIR laser for three cycles (heating−cooling curve). The rise in temperature was also monitored using a high‐resolution infrared (IR) camera (Thermal Imager Testo 875–1), and analyzed by thermography software (Testo IR Soft Software, version 3.1) [40].

### 2.8 Cellular Combinatorial Cytotoxicity Study

The cytotoxicity of DOX@FCDs and pure DOX was evaluated on human breast cancer (MCF‐7, CVCL_0031) and human lung cancer (A549, CVCL_0023) cell lines by MTT assay. Cells were seeded in a 96‐well plate (1 × 10^4^ cells per well) in complete culture medium at 37°C and 5% CO_2_ for 24 h. After 24 h, culture medium was replaced by fresh media containing different concentrations of respective systems and incubated for another 24 h at 37°C and 5% CO_2_. The relative cell viabilities were determined by the MTT assay as reported earlier [41]. Briefly, the culture medium of each well was replenished with a fresh medium having 0.5 mg/mL of MTT and further incubated for 4 h. After this, the MTT solution was removed, and the formed formazan crystals were solubilized by adding 100 μL of DMSO to each well. The absorbance of each well was measured at 570 nm on a microplate reader to calculate the cytotoxicity of the formulations. The cell viability was obtained by comparing the absorbance of the treated cells with that of control cells, which was considered as 100%. Each experiment was accomplished in triplicate, and the SD was specified in the plot. The cell viability study of FCDs NPs was also investigated to explore their biocompatibility nature in three cell lines.

In vitro photothermal toxicity was also evaluated by the MTT assay under irradiation of 980 nm laser light for prescribed time intervals. For this, cells were treated with FCDs NPs/DOX@FCDs NPs for 24 h at 37°C and 5% CO_2_. After 24 h, the culture medium was removed and cells are washed with PBS in order to remove excess of particles. After washing, cells were supplied by fresh media and irradiated for 10 min under laser power of 0.65 W at distance of 15 mm. Then, the NIR light‐treated cells were further incubated overnight at 37°C and 5% CO_2_. After the incubation period, the relative cell viabilities were determined by the MTT assay as discussed above. The morphological changes of cells after treatment at different time intervals (after incubation) were captured using a bright‐field microscope.

### 2.9 Cellular Internalization Studies by Confocal Microscopy

Cellular internalization studies were performed with MCF‐7 cells by using confocal microscopy. For imaging, cells (0.5 × 10^6^) were seeded on glass coverslips and cultured overnight. The cells were then treated with DOX and DOX@FCDs NPs (at a DOX concentration of 4 μM) for 3 and 6 h under culture conditions, followed by washing with PBS. After washing, cells were fixed with 4% para formaldehyde and permeabilized using 0.1% triton X. The cells were then mounted on a glass slide in a cell mounting medium (Invitrogen, USA) containing PI for nuclear staining and then imaged by a confocal microscopy (FV 3000, Olympus) using a green laser (488 nm) for DOX and PI and a blue laser (405 nm) for FCDs.

### 2.10 Statistics

All the data’s are presented as mean ± SD. The level of significance between each treatment group was determined using one‐way ANOVA wherever applicable. Here, *P* value of <0.05 is considered as significant, where *P < 0.05 is mentioned in the respective figure legends.

## 3 Results and Discussion

**FIGURE 1:**
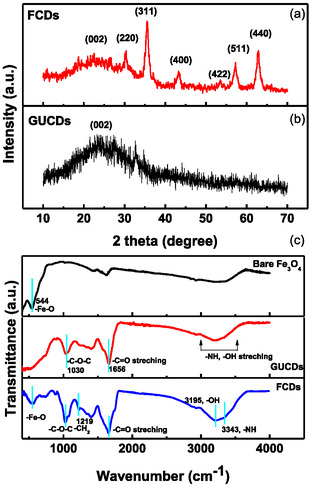
X‐ray diffraction pattern recorded for (a) GUCDs and (b) FCDs hybrid composite, (c) FTIR analysis of bare Fe_3_O_4_ NPs, GUCDs, and FCDs, respectively.

**FIGURE 2:**
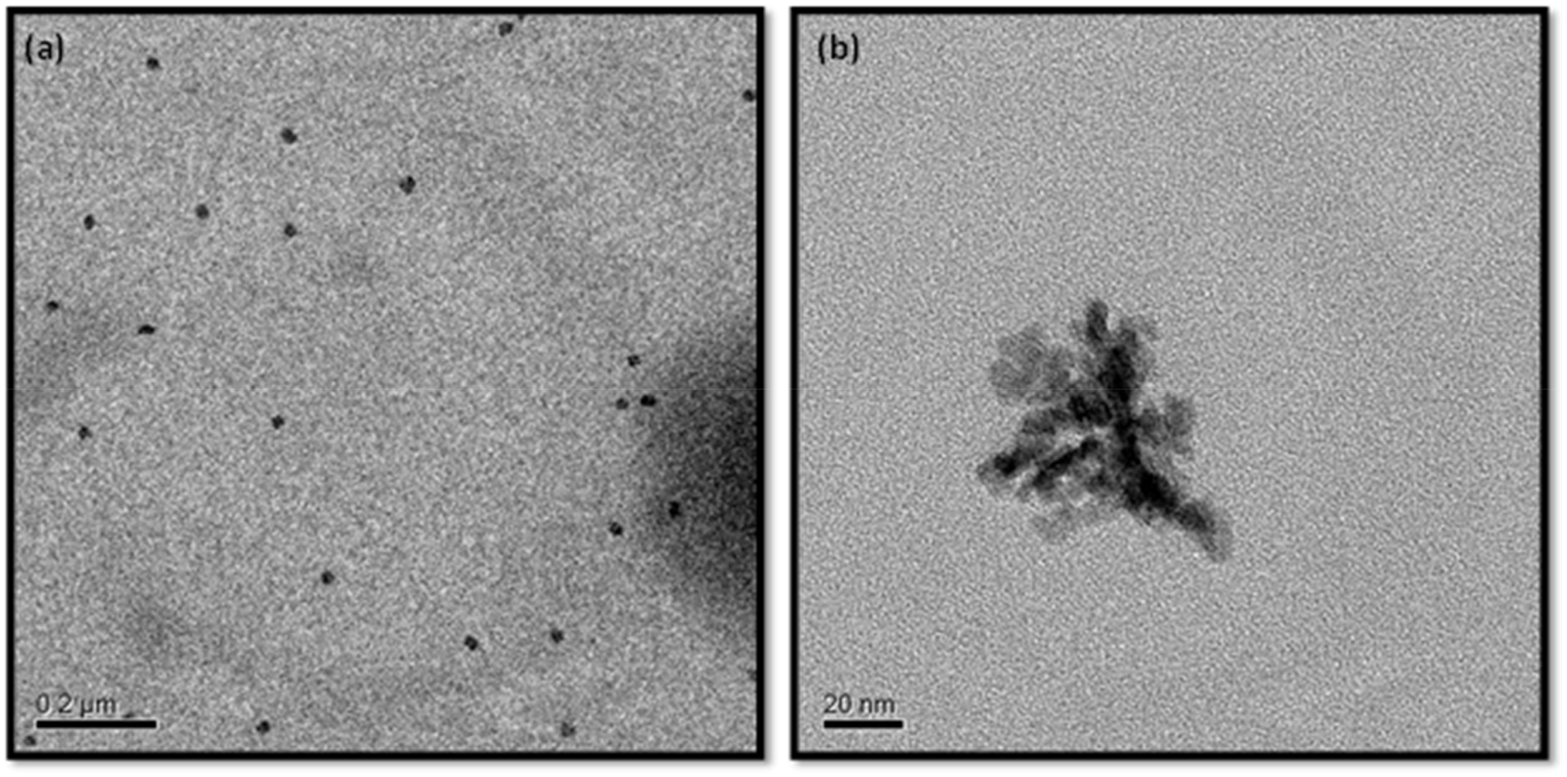
TEM images of (a) FCD and (b) FCD at high magnification.

X‐ray diffraction (XRD) measurements were carried out to realize the phase and structure of the pristine Fe_3_O_4_ nanospheres, the GUCDs carbon dots, and the GUCDs decorated Fe_3_O_4_ (FCDs) hybrid nanocomposites. The corresponding diffraction pattern of the GUCDs shows a broad diffraction peak centered at around 24.8° demonstrating the amorphous nature of the system (Figure 1b). The XRD pattern of FCDs has shown a broad shoulder centered around 2*θ* of 24° and peaks at 2*θ* values of 30.1°, 35.6°, 43.3°, 53.5°, 57.3°, 62.9° (Figure 1a). These six peaks correspond to the magnetite phase of iron oxide whereas the presence of a broad shoulder is indicative of the presence of GUCDs around the Fe_3_O_4_ NPs [42]. FTIR spectra confirmed the surface decoration of GUCDs on to the bare Fe_3_O_4_ nanoparticles. Figure 1c showed the overlay spectra for Fe_3_O_4_ NPs, GUCDs, and FCDs. The FTIR spectra for bare Fe_3_O_4_ reveal the existence of 544 cm^−1^ further approves the Fe─O bond in Fe_3_O_4_ NPs. Basic characteristic ─NH peak of urea and ─OH stretching mode from glucose is observed as a broad peak at about 3000–3450 cm^−1^. The peak at 1656 cm^−1^ is assigned to C─O stretching vibration. Stretching vibration at 1030 cm^−1^ attributed to C─O─C peak is much stronger than that of glucose and urea confirming the successful formation of N‐doped carbon dot. The surface–bound interaction between the functional moieties of two nanomaterials was shown by the minor shifting of band frequencies observed in the FTIR spectra of FCDs for the ─NH, ─C─O, and ─C─O─C stretching vibrations. The FTIR spectra of Fe_3_O_4_ coupled GUCDs (FCDs) shows the peak at 1032 cm^−1^ corresponds to C─O─C stretching, 1658 cm^−1^ correspond to ─C─O stretching and peak at 558 cm^−1^ is assigned to ─Fe─O stretching mode, giving assurance about carbon dot decorated Fe_3_O_4_ hybrid nanocomposites formation [43]. The TEM analysis of FCDs have confirmed that the size of the FCDs are of average size of 10.1 ± 1.9 nm, as shown in Figure 2a,b. The TEM images of GUCDs and bare Fe_3_O_4_ NPs were provided in Supporting Information (Supporting information, Figure S1a,b).

**FIGURE 3:**
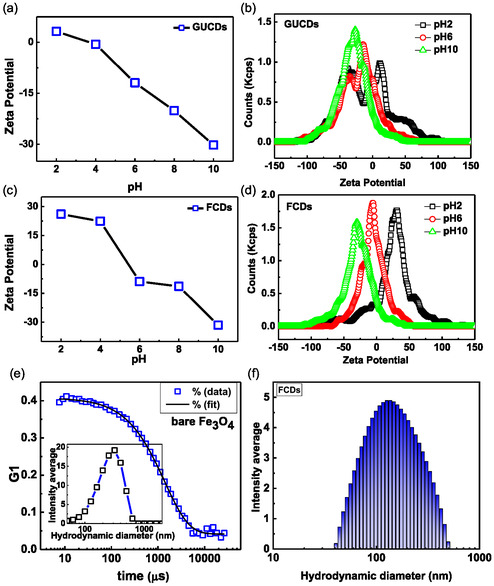
(a,c) pH‐dependent zeta potential GUCDs and FCDs NPs; (b,d) zeta potential distribution of GUCDs and FCDs at pH 2, 6, and 10, respectively. DLS autocorrelation function of (e) bare Fe_3_O_4_ and (f) FCDs; inset shows the intensity weighted average size distribution.

In order to assess the stability of the prepared colloidal nanoparticles, their surface charge features, and mean hydrodynamic diameter light scattering studies were carried out on the aqueous suspension of nanoparticles. Zeta potential of bare Fe_3_O_4_, GUCDs, and FCDs has been performed and shown in Figure 3a–d. pH‐dependent zeta potential of GUCDs have shown a steady variation of surface charge from 3.2 to −30.2 mV as shown in Figure 3a. This pH‐dependent charge reversal is mainly due to the ionizable hydroxyl and amine groups present at the surface of the carbon dots. The zeta potential distribution of GUCDs at pH 2, 6, and 10 have been shown in Figure 3b with an average surface potential of 3.2, −11.9, and −30.2 mV respectively. As the carbon dots are decorated on to the surface of the bare Fe_3_O_4_ nanoparticles, same type of variation in zeta potential is also observed in FCDs (Figure 3c,d). In FCDs the zeta potential has varied from 26.1 to −31.5 mV in pH 2–10, respectively, as shown in Figure 3d. Zeta potential of bare Fe_3_O_4_ in nano pure water has been added in Supporting Information (Figure S2, Table ST1). The negative zeta potential at −12 mV at physiological makes them colloidally stable and ideal for therapeutic application [44]. Figure 3e and f showed the DLS autocorrelation function of bare Fe_3_O_4_ and FCDs, respectively. It has been observed that the autocorrelation function decays quickly as a function of time and the mean intensity weighted hydrodynamic (D_h_) diameter was found to be around 151.3 ± 5.2 and 165.5 ± 12.5 with polydispersity of 0.3 and 0.2, respectively. The number‐average size distribution of FCDs was found to be 85.3 nm from intensity‐weighted diameter by using correlation software and Stokes–Einstein equation. The polydispersity of particles contributes to the observed larger size of particles in DLS. Further, at 40,000 Oe, FCDs show superparamagnetic behavior with a maximum magnetization of 33.5 emu/g (Supporting information, Figure S3). The observed magnetization of FCDs was found to be lower than the bare Fe_3_O_4_ nanoparticles 67.6 emu/g, prepared by Jerina et al. [45]. The existence of non‐magnetic carbon dot decoration on the surface of Fe_3_O_4_ nanoparticles (FCDs) is further supported by this decrease in magnetization.

### 3.1 Optical Property Analysis

**FIGURE 4:**
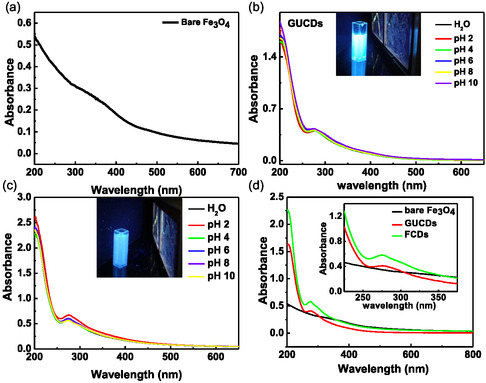
UV–visible spectra of (a) bare iron oxide nanoparticles, (b) GUCDs at different pH, inset shows the highly intense blue emission under UV irradiation, (c) FCDs at different pH, inset shows the highly intense blue emission under UV irradiation and (d) comparison spectra of bare Fe_3_O_4_, GUCDs, and FCDs.

The absorption spectra of the bare Fe_3_O_4_ were taken in Figure 4a. After dialysis of the crude solution against ultrapure water, the solution of GUCDs was clear and light brown in color under ambient light and produced bright blue fluorescence under UV light, as shown in inset of Figure 4b. The UV absorption spectra of GUCDs showed a broad absorption band in the range of 200–550 nm. The peaks highlighted at 206 and 276 nm in Figure 4b arise due to the π−π∗ and n–π∗ electronic transition associated with sp^2^ hybridized ─C─C and carbonyl groups, respectively. The weak shoulder peak centered at about 206 nm corresponds to π−π∗ transition of the aromatic sp^2^ domains and produces no detectable PL [46, 47]. The GUCDs decorated FCDs also showed bright blue emission upon UV irradiation with the characteristic absorption peak at 275 nm (Figure 4c). The observed bright and colorful luminescence emissions of FCDs thus must be due to the surface‐passivated carbon dot, i.e., GUCDs. The pH‐dependent UV–vis spectra also showed no significant change in their features, essentially as shown in both Figure 4b and c. The spectral feature of bare Fe_3_O_4_, GUCDs, and FCDs was depicted in Figure 4d.

**FIGURE 5:**
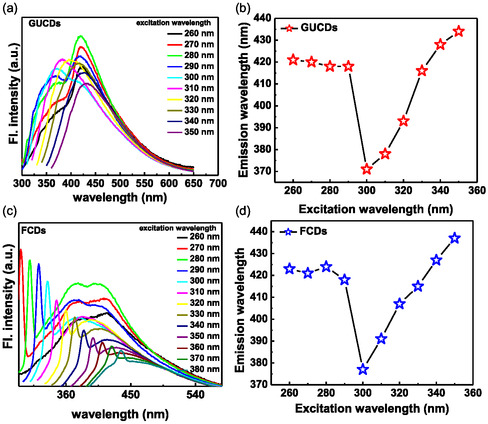
Excitation dependent fluorescence emission spectra of (a) GUCDs, (c) FCDs, excitation versus emission wavelength graph for (b) GUCDs and (d) FCDs, respectively.

Figure 5a,c showed the excitation dependent emission spectra of GUCDs and FCDs, respectively, where the maximum emission was found upon excitation with 280 nm for both GUCDs and FCDs, respectively. Thus far, electronic conjugate structures and emissive traps have been postulated as the two most common fluorescence processes of CDs. The mechanism behind carbon dots’ PL behavior is quite intricate and hasn’t been thoroughly documented yet. The distribution of the various surface energy traps of the carbon dots and the existence of varying particle sizes are the likely causes of the PL behavior [48, 49, 50]. The size fluctuation of the carbon dots is the cause of the variation in the emission peak’s position. The quantum confinement effect, similar to that of semiconductor quantum dots, causes the energy gap to widen as the size of the carbon dots decreases and vice versa. As a result, smaller particles are excited at lower wavelengths while bigger particles are stimulated at higher wavelengths. The quantity of particles excited at a specific wavelength determines the intensity of fluorescence. As the largest number of particles were excited at 280 nm, the highest PL intensity of carbon dots was seen at that excitation wavelength. Also, due to the presence of different functional groups on the surface of carbon dots, large number of emissive traps could also be produced and contribute towards the excitation dependent emission maxima shifting. On illuminating the carbon dots at a certain excitation wavelength, corresponding surface state emissive traps become dominant. The correlation between the excitation and emission wavelengths in GUCDs and FCDs was shown in Figure 5b,d, respectively. Both GUCDs and FCDs have shown excitation dependent emission maxima shift. The FCDs have shown maximum emission at 400 nm upon excitation with 280 nm light. The quantum yield of FCDs was calculated and found to be 3.13% using relative method using tryptophan as standard. The detailed calculation procedure and spectra were provided in Supporting Information (Supporting information, Figure S4). Conversely, when the specified excitation wavelengths were less than 300 nm, the emission peaks were blueshifted. Electrons can be stimulated to a higher energy level thanks to the distinct absorption energy levels that are produced from the formed graphitic core [51, 52]. Through radiative recombination, the excited electrons might be efficiently relaxed to the ground state, resulting in a shorter emission wavelength. The pH‐dependent emission spectra of both GUCDs and FCDs were produced in Supporting Information (Supporting Information S5 and S6). Both the emissive nanosystems have shown excitation‐dependent emission spectra in all five pH variations, with maximum emission at 280 nm wavelength. The irradiation stability of the synthesized system was also checked by irradiating the FCDs sample solution under Xe lamp for 2 h. The PL spectra have not shown any significant change in the emission intensity while kept at 280 nm excitation. The fluorescence characteristics of the produced carbon dots is therefore encouraging for their various potential uses. Because the PL intensity is sensitive to the carbon dot concentration, carbon dots can be used in biological detection at low concentrations. A comparative table of optical properties have been provided here below (Table 1).

**TABLE 1:** A comparative table of optical properties.

Sl. No.	Precursor	Synthetic route	QY%	Ex/Em, nm	Magnetization value (emu/gm)	Ref.
1.	Streptomycin	Hydrothermal, 200°C, 12 h	7.6%	335/410	—	[53]
2.	Oolong tea	Hydrothermal, 220°C, 3 h	4.9%	470/518	—	[54]
3.	Polyvinylpyrrolidon	Hydrothermal, 200°C, 6 h	6%	380/450	—	[55]
4.	Tartaric acid and alkylol amines	Microwave, 650 W, 7 min	6.4%–1.8%	340/440 360/450	—	[56]
5.	Glycerol and ethylenediamine	Microwave, 1000 W, 20 min	7.5%	360/429	—	[57]
6.	Fe_3_O_4_@CDs	Hydrothermal	Not available	345/440	54.1	[58]
7.	Fe_3_O_4_@CDs	Hydrothermal	Not available	visible/510	38.1	[59]
8.	Fe_3_O_4_@CDs	Two step process	Not available	300/413	40 emu/g	[60]
9.	Fe_3_O_4_@CDs	Two step process	Not available	UV/420	34 emu/g	[61]
10.	Fe_3_O_4_@Tyr.	Two step process	4%	Emission @375 nm	35 emu/g	[62]
11.	FCDs	Two step process	3.1%	280/400	33.5	This work

### 3.2 Chemo‐Photothermal Efficacy

**FIGURE 6:**
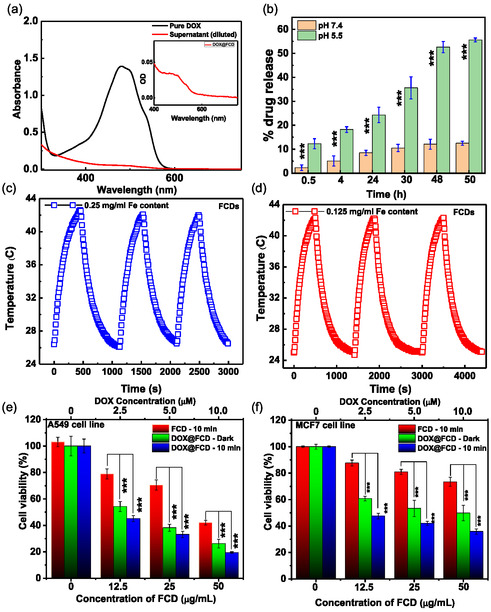
(a) UV–vis spectra of supernatant after removal of drug loaded FCDs and pure DOX, respectively, (b) drug release pattern of DOX@FCDs, laser power dependent temperature profile of FCDs at power 0.9 W over three cycles and in two different concentration (c) 0.25 mg/ml and (d) 0.125 mg/ml., Cell viability of nanoformulation via MTT of in (e) A549 and (f) MCF7 cell lines after 24 h incubation. Data represent the mean ± SD (*n* = 3); the statistically significant values were obtained using a student t‐test, ***p < 0.001.

In order to explore the use of FCDs as carriers for delivery of chemotherapeutic drugs, their drug loading behavior using DOX was investigated. Around 22% of drug loading efficiency was observed at 1:10 ratio. The typical absorption spectra of DOX, supernatant solution and washing are shown in Figure 6a, which confirms the loading capacity (absorbance intensity of supernatant solution obtained after removal of DOX‐FCDS significantly reduced as compared to pure DOX). The inset are shown in Figure 6a reassured the presence of DOX by the presence of hump at around 485 nm in UV spectra of DOX@FCDs dispersion after free drug separation [63]. The drug loading was further reconfirmed using fl. Spectroscopy. The DOX@FCDs were excited at 280 and 485 nm which showed the emission corresponding to the bare FCDs and DOX at 400 and 588 nm, respectively (Supporting information, Figure S7a). This finding was further corroborated with emission of pure DOX molecule in water medium. Upon excitation at 485 nm, free doxorubicin (DOX) exhibited a characteristic emission maximum at 592 nm, whereas the DOX‐loaded carbon dots (DOX@FCDs) showed a slightly blueshifted emission at 588 nm along with an evident broadening of the fluorescence band (Supporting information, Figure S7b). The minor blue shift can be attributed to the change in the local microenvironment of DOX upon interaction with the carbon dot surface. In free solution, DOX molecules are surrounded by a highly polar aqueous medium, while after adsorption onto the FCDs, they experience a relatively less polar and more hydrophobic environment, leading to stabilization of the ground state and hence a blue shift in emission. In addition, π–π stacking and electrostatic interactions between the aromatic moieties of DOX and the graphitic domains or surface functional groups of FCDs can influence the electronic energy levels of DOX, resulting in the observed spectral shift. The increase in emission bandwidth further indicates a heterogeneous microenvironment around the loaded DOX molecules, arising from diverse binding orientations and interaction strengths on the FCD surface. These changes collectively confirm the successful loading of DOX onto the carbon dots and the alteration of its photo physical properties due to nanoscale surface interactions [64, 65].

After the formation drug‐loaded nanoformulation, the release profile of DOX at 37°C under reservoir‐sink condition (reservoir: pH 5.5, 7.4 and sink: pH 5.5, 7.4, respectively) was carried out (Figure 6b). The release study has suggested that the loaded drug molecules release slowly over a period of 48 h. The shape of the release plot indicates that the complete release of DOX was not even attained in 50 h. It has been observed that about 12 and 55% of loaded DOX molecules were released from the DOX@FCDs system at pH 7.4 and pH 5.5, respectively. The selection of pH 5.5 in the in vitro DOX release study was intentional to mimic the endosomal and lysosomal microenvironments encountered after nanoparticle internalization by cancer cells, rather than the extracellular tumor milieu. While the tumor extracellular pH typically ranges from 6.5–6.8, the intracellular compartments involved in nanoparticle trafficking, such as endosomes (pH≈6.0) and lysosomes (pH≈4.5–5.5), are considerably more acidic [8]. The pH 5.5 condition was therefore chosen to simulate intracellular drug release behavior, reflecting how FCDs might release DOX once internalized and exposed to acidic vesicular environments. In contrast, the pH 7.4 condition represents physiological blood and extracellular conditions. Figure 6c and d displays the concentration‐dependent temperature elevation study of an aqueous solution of FCDs as a function of NIR laser power (continuous wavelength laser, 980 nm, 0.9 W. Although the final equilibrium temperature in Figure 6c,d appears similar (approximately 42.5°C), the time required to reach this temperature varied significantly with concentration, confirming that the photothermal response is indeed concentration‐dependent. The time required to reach 42.5^o^C in 0.9 W are 450 and 500 s, respectively, for two FCDs dispersion containing 0.25 and 0.125 mg/ml of Fe, showing the concentration dependent heating of the nanoparticles whereas the water is only heated up to 35.1^o^C even after 600 s. The photothermal stability and reusability of the FCDs NPs was observed up to 3 cycle (heating–cooling curve) which has shown extreme stability along with pertinent heating efficacy. At higher nanoparticle concentrations, the rate of temperature rise was markedly faster, indicating enhanced photothermal conversion efficiency due to greater NIR absorption and heat generation. In contrast, lower concentrations required longer irradiation times to achieve the same terminal temperature.

**FIGURE 7:**
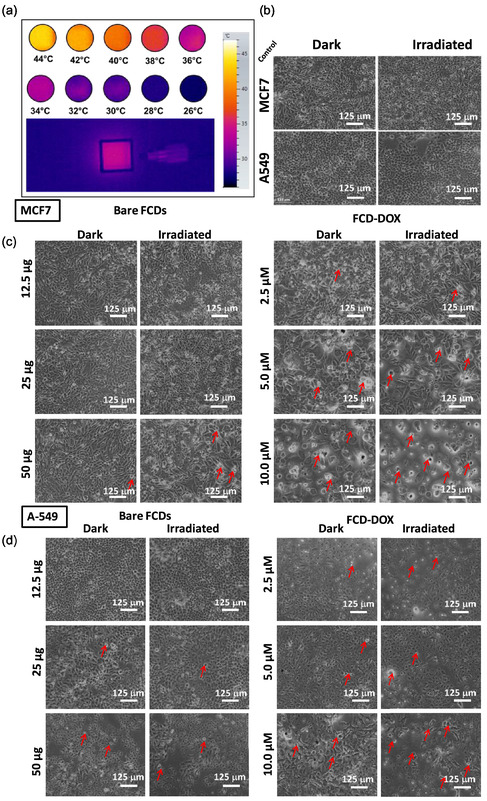
(a) IR thermograms showing increase in temperature of aqueous suspension of FCDs NPs under 980 nm laser light irradiation, (b–d) bright‐field microscopy images of A549 and MCF‐7 cells without treatment and after different treatments with FCDs and FCD‐DOX NPs at 24 hr in different concentration. Red arrows indicate cells depicting apoptotic morphology.

The biocompatibility of FCDs NPs toward normal (WI26VA4) and cancer (A549 and MCF‐7) cell lines was investigated under culture condition at 37°C by the MTT assay (Supporting Information, Figure S8). It has been found that more than 95% of WI26VA4 cells were viable even after 24 h treatment with 12.5 μg/mL of FCDs NPs. This suggested the biocompatibility nature of FCDs NPs. However, they exhibited dose‐dependent toxicity at higher concentrations (beyond 50 μg/mL). Further, to investigate the combinatorial chemo‐photothermal efficiency of FCDs NPs, DOX‐loaded FCDs NPs (DOX@FCD) toward A549 and MCF‐7 cells were also explored in the presence and absence of NIR light. Figure 6 shows the viability of (e) A549 and (f) MCF‐7 cells upon 24 h of incubation with FCDs NPs and DOX@FCD NPs followed by 10 min of irradiation of NIR light (L‐10) and dark (D) conditions. It was found that the cell killing efficacy of the DOX@FCD NPs was bettered in combination with NIR irradiation rom 74.2% to 81.4% in A549 and 51.2% to 74.2% in MCF7 cell lines respectively than the DOX@FCD NPS alone which signifying the combination of chemotherapy and photothermal therapy is more effective than the individual treatment of chemotherapy or PTT [40]. The color change from deep blue (low temperature) to bright yellow (high temperature) shown in IR thermogram also providing the evidence of heating ability of the FCDs NPs in aqueous dispersion of 125 μg/mL of Fe (0.9 W) (Figure 7a). Moreover, the temperature profile confirmed the localized of the photothermal heating effect of the FCDs which will further reduce the unwanted side effect on surrounding healthy cells while treating in vivo*.* This cell killing effect was even more pronounced and supported by the cellular morphology investigation upon different treatment and shown in Figure 7b–e. The observed higher toxicity due to the chemo‐photothermal effect is due to enhanced sensitivity of cancer cells towards DOX along with NIR irradiation. The red arrows are showing the apoptotic morphology of the cancer cells treated with DOX@FCDs at various concentration with laser irradiation.

**FIGURE 8:**
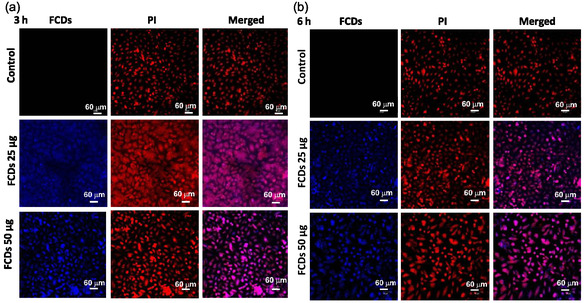
Confocal microscopy images of MCF‐7 cells after incubation with FCD NPs under culture conditions at two different time frame (a) 3 and (b) 6 h (PI was used for nuclear staining; red filter for PI and blue filter for FCDs).

The intracellular delivery of the drug‐loaded system is important for achieving higher therapeutic efficacy. Thus, we also explored the cellular uptake of FCDs NPs and DOX@FCDs NPs in cancer (MCF‐7) cells by confocal microscopy. Figure 8a,b shows the confocal microscopy images of cancer cells after incubation with FCDs NPs at two different concentrations, 25 and 50 µg/ml, at two different time frames (3 and 6 h). The blue color fluorescence emission arises from carbon dot decorated on the surface of iron oxide core (FCDs) while irradiating with 405 nm laser, whereas the red emission of PI (laser excitation 488 nm) is coming from the nucleus. The magenta color emission originates from the merged image of FCDs and PI fluorescence, suggesting that FCDs NPs are internalized inside the cell in a time and concentration‐dependent manner (Figure 8a,b). Thus, these nanohybrids can be an excellent bio‐imaging agent for cellular labeling. Furthermore, the cellular internalization of DOX@FCDs showed major internalization in cytoplasm as well as with increasing the concentration in nucleus (Supporting information S9). It is believed that the DOX‐loaded NPs were first taken up by passive targeting, i.e., enhanced permeability and retention into the cytoplasm, followed by slow entry inside the nucleus via endocytosis pathway, and hence showing time‐dependent increase of fluorescence signal intensity in FCDs.

## 4 Conclusion

In summary, a highly water‐dispersible luminescent nanohybrid is a soft‐chemical approach. The introduction of carbon dots onto the surface of Fe_3_O_4_ NPs not only provides luminescence, but the magnetic core also presents means of ease in separation of NPs after cellular labeling for bio‐imaging applications. The successful formation of the FCDs was evident from XRD, TEM, fluorescence, FTIR, and light scattering measurements. The aqueous suspension of FCDs was found to be NIR active and showed excellent heating efficacy under irradiation with a 980 nm laser. Moreover, these FCDs NPs were conjugated with positively charged anticancer drug, DOX through electrostatic interaction. The DOX@FCDs systems showed substantial cellular internalization and revealed a pH‐dependent release behavior. From in vitro studies, it has been found that luminescent FCDs nanohybrids showed time and concentration dependent cellular imaging efficacy. Moreover, the DOX‐loaded system exhibited a potential application in combinatorial chemo‐photothermal efficiency via apoptotic induction with much higher toxicity towards cancer cells upon NIR irradiation.

## Author Contributions

B. D. contributed to conceptualization, methodology, formal analysis, investigation, writing – original draft, review and editing and supervision; S. G., S. P., and P. B. contributed to methodology, R. B. contributed to methodology; N. K. P. contributed to methodology, K. C. B. contributed to editing and supervision, P. A. H. supervision.

## Supporting Information

Additional supporting information can be found online in the Supporting Information Section.

## Conflicts of Interest

The authors declare no conflicts of interest.

## Supporting information

Supplementary Material

## Data Availability

All data generated or analyzed during this study are included in this published article (and its supplementary information files).

## References

[1] M. Adeel , F. Duzagac , V. Canzonieri , and F. Rizzolio , “Self‐Therapeutic Nanomaterials for Cancer Therapy: A Review,” Acs Applied Nano Materials 3, no. 6 (2020): 4962–4971.

[2] S. Basu , P. Biswas , M. Anto , et al., “Nanomaterial‐Enabled Drug Transport Systems: A Comprehensive Exploration of Current Developments and Future Avenues in Therapeutic Delivery,” Biotechnology 14 (2024): 289. 10.1007/s13205-024-04135-y PMC11534931 39507057

[3] J. Cardellini , A. Surpi , B. Muzzi , et al., “Magnetic–Plasmonic Nanoscale Liposomes with Tunable Optical and Magnetic Properties for Combined Multimodal Imaging and Drug Delivery,” Acs Applied Nano Materials 7, no. 4 (2024): 3668–3678.

[4] B. Dutta , K. C. Barick , P. A. Hassan , and A. K. Tyagi , “Recent Progress and Current Status of Surface Engineered Magnetic Nanostructures in Cancer Theranostics,” Advances in Colloid and Interface Science. (2024): 103320. 39515063 10.1016/j.cis.2024.103320

[5] Z. Kang and S. T. Lee , “Carbon Dots: Advances in Nanocarbon Applications,” Nanoscale 11, no. 41 (2019): 19214–19224. 31513215 10.1039/c9nr05647e

[6] W. Su , H. Wu , H. Xu , et al., “Carbon Dots: A Booming Material for Biomedical Applications,” Materials Chemistry Frontiers 4, no. 3 (2020): 821–836.

[7] B. Rezaei , P. Yari , S. M. Sanders , et al., “Magnetic Nanoparticles: A Review on Synthesis, Characterization, Functionalization, and Biomedical Applications,” Small 20, no. 5 (2024): 2304848. 10.1002/smll.202304848 37732364

[8] B. Dutta , K. C. Barick , and P. A. Hassan , “Recent Advances in Active Targeting of Nanomaterials for Anticancer Drug Delivery,” Advances in Colloid and Interface Science 296 (2021): 102509. 34455211 10.1016/j.cis.2021.102509

[9] H. Wang , Y. Sun , J. Yi , et al., “Fluorescent Porous Carbon Nanocapsules for Two‐Photon Imaging, NIR/pH Dual‐Responsive Drug Carrier, and Photothermal Therapy,” Biomaterials 53 (2015): 117–126. 25890712 10.1016/j.biomaterials.2015.02.087

[10] L. Lin , Z. Bao , P. Jiang , et al., “Superior Biocompatible Carbon Dots for Dynamic Fluorescence Imaging of Nucleoli in Living Cells,” Biomaterials Science 11, no. 8 (2023): 2935–2949. 36912088 10.1039/d2bm02139k

[11] T. Ghosh , S. Nandi , S. K. Bhattacharyya , et al., “Nitrogen and Sulphur Doped Carbon Dot: An Excellent Biocompatible Candidate for in‐Vitro Cancer Cell Imaging and beyond,” Environmental Research 217 (2023): 114922. 36435492 10.1016/j.envres.2022.114922

[12] B. Dutta , A. Nema , N. G. Shetake , et al., “Glutamic Acid‐Coated Fe3O4 Nanoparticles for Tumor‐Targeted Imaging and Therapeutics,” Materials Science and Engineering: C 112 (2020): 110915. 32409067 10.1016/j.msec.2020.110915

[13] B. Dutta , N. G. Shetake , B. K. Barick , et al., “PH Sensitive Surfactant‐Stabilized Fe3O4 Magnetic Nanocarriers for Dual Drug Delivery,” Colloids and Surfaces B: Biointerfaces 162 (2018): 163–171. 29190467 10.1016/j.colsurfb.2017.11.054

[14] B. Dutta , S. B. Shelar , A. Nirmalraj , et al., “Smart Magnetic Nanocarriers for Co‐Delivery of Nitric Oxide and Doxorubicin for Enhanced Apoptosis in Cancer Cells,” ACS Omega 8, no. 47 (2023): 44545–44557. 38046289 10.1021/acsomega.3c03734 PMC10688159

[15] B. Dutta , N. G. Shetake , S. L. Gawali , et al., “PEG Mediated Shape‐Selective Synthesis of Cubic Fe3O4 Nanoparticles for Cancer Therapeutics,” Journal of Alloys and Compounds 737 (2018): 347–355.

[16] B. Dutta , S. B. Shelar , K. C. Barick , et al., “Surface Engineered Fe3O4 Nanomagnets for pH‐Responsive Delivery of Gemcitabine Hydrochloride and In Vivo Tracking by Radiolabeling,” Materials Advances 4, no. 1 (2023): 195–204.

[17] T. N. Edirisuriya , T. M. S. U. Gunathilake , Y. C. Ching , and H. Noothalapati , “Curcumin Targeted Drug Delivery Using Iron Oxide Nanoparticle Incorporated Magnetic Responsive Carboxymethyl Cellulose Hydrogel,” Polymer Science Series B. (2024): 1–14.

[18] A. Shakeri-Zadeh and J. W. Bulte , “Imaging‐Guided Precision Hyperthermia with Magnetic Nanoparticles,” Nature Reviews Bioengineering (2024): 1–16. 10.1038/s44222-024-00257-3 PMC12011369 40260131

[19] M. Torkpur‐Biglarianzadeh and M. Salami‐Kalajahi , “Multilayer Fluorescent Magnetic Nanoparticles with Dual Thermoresponsive and pH‐Sensitive Polymeric Nanolayers as Anti‐Cancer Drug Carriers,” RSC Advances 5 (2015): 29653–29662.

[20] K. Sato , E. Abe , M. Takahashi , and J.‐i. Anzai , “Loading and Release of Fluorescent Dye from Layer‐by‐Layer Film‐Coated Magnetic Particles in Response to Hydrogen Peroxide,” Journal of Colloid and Interface Science 432 (2014): 92–97. 25084230 10.1016/j.jcis.2014.06.039

[21] S. Wang , W. Yang , H. Du , et al., “Multifunctional Reduction‐Responsive SPIO&DOX‐Loaded PEGylated Polymeric Lipid Vesicles for Magnetic Resonance Imaging‐Guided Drug Delivery,” Nanotechnology 27 (2016): 165101. 26941226 10.1088/0957-4484/27/16/165101

[22] E. Perillo , K. Hervé‐Aubert , E. Allard‐Vannier , A. Falanga , S. Galdiero , and I. Chourpa , “Synthesis and In Vitro Evaluation of Fluorescent and Magnetic Nanoparticles Functionalized with a Cell Penetrating Peptide for Cancer Theranosis,” Journal of Colloid and Interface Science 499 (2017): 209–217. 28388503 10.1016/j.jcis.2017.03.106

[23] B. Ullrich , J. S. Wang , X. Y. Xiao , and G. J. Brown , “Quantum Dots and Nanostructures: Synthesis, Characterization, and Modeling IX,” International Society for Optics And Photonics 2012): 82710.

[24] A. Armaselu , “Determination of Quantum Dot Size by Fourier Transform Visible Spectroscopy,” Optoelectronics and Advanced Materials, Rapid Communications 9 (2015): 531–536.

[25] J. Wang , S. Han , D. Ke , and R. Wang , “Semiconductor Quantum Dots Surface Modification for Potential Cancer Diagnostic and Therapeutic Applications,” Journal of Nanomaterials 1 (2012): 129041.

[26] J. Sobhanan , J. V. Rival , A. Anas , E. S. Shibu , Y. Takano , and V. Biju , “Luminescent Quantum Dots: Synthesis, Optical Properties, Bioimaging and Toxicity,” Advanced Drug Delivery Reviews 197 (2023): 114830. 37086917 10.1016/j.addr.2023.114830

[27] C. Bai and M. Tang , “Progress on the Toxicity of Quantum Dots to Model Organism‐Zebrafish,” Journal of Applied Toxicology 43, no. 1 (2023): 89–106. 35441386 10.1002/jat.4333

[28] Y. Yao , T. Zhang , and M. Tang , “The DNA Damage Potential of Quantum Dots: Toxicity, Mechanism and Challenge,” Environmental Pollution 317 (2023): 120676. 36395913 10.1016/j.envpol.2022.120676

[29] W. Wang , X. Liu , X. Li , B. Geng , and E. Zhao , “Application of MRI Imaging Technology Based on Magnetic Nanoparticles in Diagnosis and Prognosis Evaluation of Prostate Cancer,” SLAS Technology 29, no. 6 (2024): 100225. 39581264 10.1016/j.slast.2024.100225

[30] J. Chen , Z. Guo , H. Wang , et al., “Multifunctional Fe3O4@ C@ Ag Hybrid Nanoparticles as Dual Modal Imaging Probes and Near‐Infrared Light‐Responsive Drug Delivery Platform,” Biomaterials 34 (2013): 571. 23092859 10.1016/j.biomaterials.2012.10.002

[31] H. Zhu , J. Tao , W. Wang , et al., “Magnetic, Fluorescent, and Thermo‐Responsive Fe3O4/Rare Earth Incorporated Poly (St‐NIPAM) Core–shell Colloidal Nanoparticles in Multimodal Optical/Magnetic Resonance Imaging Probes,” Biomaterials 34 (2013): 2296. 23274069 10.1016/j.biomaterials.2012.11.056

[32] X. Wang , L. Cao , S. Yang , et al., “Bandgap‐Like Strong Fluorescence in Functionalized Carbon Nanoparticles,” Angewandte Chemie International Edition 49 (2010): 5310. 20572221 10.1002/anie.201000982 PMC3511838

[33] L. Cheng , K. Yang , Y. Li , et al., “Multifunctional Nanoparticles for Upconversion Luminescence/MR Multimodal Imaging and Magnetically Targeted Photothermal Therapy,” Biomaterials 33 (2012): 2215. 22169825 10.1016/j.biomaterials.2011.11.069

[34] A. A. Lipengolts , Y. A. Finogenova , V. A. Skribitsky , et al., “CT and MRI Imaging of Theranostic Bimodal Fe3O4@ Au Nanoparticles in Tumor Bearing Mice,” International Journal of Molecular Sciences 24, no. 1 (2022): 70. 36613511 10.3390/ijms24010070 PMC9820463

[35] B. Muzzi , M. Albino , A. Gabbani , et al., “Star‐Shaped Magnetic‐Plasmonic Au@ Fe3O4 Nano‐Heterostructures for Photothermal Therapy,” ACS Applied Materials & Interfaces 14, no. 25 (2022): 29087–29098. 35708301 10.1021/acsami.2c04865 PMC9247976

[36] L. Lin , L. Chen , J. Yan , J. Yan , J. Du , et al., “Advances of Nanoparticle‐Mediated Diagnostic and Therapeutic Strategies for Atherosclerotic Plaques: A Focus on Multimodal Imaging and Theranostics,” Frontiers in Bioengineering and Biotechnology 11 (2023): 1268428. 38026849 10.3389/fbioe.2023.1268428 PMC10666776

[37] M. M. Koç , U. Paksu , N. Kurnaz Yetim , B. Coşkun , E. Hasanoğlu Özkan , et al., “Nanoparticles in Photothermal Therapy‐Based Medical and Theranostic Applications: An Extensive Review,” European Physical Journal Plus 140 (2025): 514.

[38] B. Dutta , N. G. Shetake , S. Patra , et al., “pH‐Responsive Magnetic Nanocarriers for Chelator‐Free Bimodal (MRI/SPECT‐CT) Image‐Guided Chemo‐Hyperthermia Therapy in Human Breast Carcinoma,” Journal of Materials Chemistry. B 12, no. 45 (2024): 11759–11777. 39417226 10.1039/d4tb00980k

[39] H. Wang , J. Shen , Y. Li , et al., “Magnetic Iron Oxide–fluorescent Carbon Dots Integrated Nanoparticles for Dual-Modal Imaging, Near‐Infrared Light‐Responsive Drug Carrier and Photothermal Therapy,” Biomaterials Science 2, no. 6 (2014): 915–923. 32481822 10.1039/c3bm60297d

[40] S. Gupta , B. Dutta , S. B. Shelar , et al., “Polyphosphate‐Mediated Crystallographic and Colloidal Stabilization of CuS Nanoparticles: Enhanced NIR‐Responsive Chemo‐Photothermal Efficacy,” Acs Applied Bio Materials 7, no. 10 (2024): 6641–6655. 10.1021/acsabm.4c00838 39257063

[41] A. Gangwar , S. Gupta , J. Gupta , et al., “Growth of Dendritic CuS Nanostructures for Photoacoustic Image Guided Chemo‐Photothermal Therapy,” Journal of Photochemistry and Photobiology A: Chemistry 459 (2025): 116084.

[42] B. Dutta , S. B. Shelar , V. Rajan , et al., “Gelatin Grafted Fe3O4 Based Curcumin Nanoformulation for Cancer Therapy,” Journal of Drug Delivery Science and Technology 67 (2022): 102974.

[43] M. W. Abbas , R. A. Soomro , N. H. Kalwar , et al., “Carbon Quantum Dot Coated Fe3O4 Hybrid Composites for Sensitive Electrochemical Detection of Uric Acid,” Microchemical Journal 146 (2019): 517–524.

[44] K. Radhakrishnan and P. Panneerselvam , “Green Synthesis of Surface‐Passivated Carbon Dots from the Prickly Pear Cactus as a Fluorescent Probe for the Dual Detection of Arsenic(III) and Hypochlorite Ions from Drinking Water,” Rsc Advances 8, no. 53 (2018): 30455–30467. 35546865 10.1039/c8ra05861j PMC9085518

[45] J. Majeed , K. C. Barick , N. G. Shetake , B. N. Pandey , P. A. Hassan , and A. K. Tyagi , “Water‐Dispersible Polyphosphate‐Grafted Fe_3_O_4_ Nanomagnets for Cancer Therapy,” Rsc Advances 5 (2015): 86754–86762.

[46] N. Dhenadhayalan , K.-C. Lin , R. Suresh , and P. Ramamurthy , “Unravelling the Multiple Emissive States in Citric‐Acid‐Derived Carbon Dots,” The Journal of Physical Chemistry C 120 (2016): 1252–1261.

[47] T. N. J. I. Edison , R. Atchudan , M. G. Sethuraman , J.‐J. Shim , and Y. R. Lee , “Microwave‐Assisted Green Synthesis of Fluorescent N‐Doped Carbon Dots: Cytotoxicity and Bio‐Imaging Applications,” Journal of Photochemistry and Photobiology B: Biology 161 (2016): 154–161. 27236237 10.1016/j.jphotobiol.2016.05.017

[48] S. N. Baker and G. A. Baker , “Luminescent Carbon Nanodots: Emergent Nanolights,” Angewandte Chemie International Edition 49 (2010): 6726–6744. 20687055 10.1002/anie.200906623

[49] S. Sahu , B. Behera , T. K. Maiti , and S. Mohapatra , “Simple One‐Step Synthesis of Highlyb Luminescent Carbon Dots from Orange Juice: Application as Excellent Bio‐Imaging Agents,” Chemical Communications 48 (2012): 8835–8837. 22836910 10.1039/c2cc33796g

[50] B. Dutta , A. Waghmare , S. K. Das , et al., “Fluorescence Tunable Carbon Dots for In Vitro Nuclear Dynamics and Gastrointestinal Imaging in Live Zebrafish and Their In Vivo Toxicity Evaluation by Cardio‐Craniofacial Dysfunction Assessment,” Nanoscale 17 (2025): 4502. 39801425 10.1039/d4nr04077e

[51] M.‐H. Jang , S. H. Song , H. D. Ha , T. S. Seo , S. Jeon , and Y.‐H. Cho , “Origin of Extraordinary Luminescence Shift in Graphene Quantum Dots with Varying Excitation Energy: An Experimental Evidence of Localized sp^2^ Carbon Subdomain,” Carbon 118 (2017): 524–530.

[52] Y. Zhang , Z. L. Yuan , A. Yu , and S. H. Yu , “Selective Detection of Ferric Ions by Blue‐Green Photoluminescent Nitrogen‐Doped Phenol Formaldehyde Resin Polymer,” Small 10 (2014): 3662–3666. 24863556 10.1002/smll.201303461

[53] W. Wang , Y.‐C. Lu , H. Huang , J.‐J. Feng , J.‐R. Chen , and A.‐J. Wang , “Facile Synthesis of Water‐Soluble and Biocompatible Fluorescent Nitrogen‐Doped Carbon Dots for Cell Imaging,” The Analyst 139 (2014): 1692–1696. 24551871 10.1039/c3an02098c

[54] L. Shi , B. Zhao , X. Li , et al., “Green‐Fluorescent Nitrogen‐Doped Carbon Nanodots for Biological Imaging and Paper‐Based Sensing,” Analytical Methods 9 (2017): 2197–2204.

[55] I. Milenkovic , M. Algarra , C. Alcoholado , et al., “Fingerprint Imaging Using N‐Doped Carbon Dots,” Carbon 144 (2019): 791–797.

[56] M. Xu , S. Xu , Z. Yang , et al., “Hydrophilic and Blue Fluorescent N‐Doped Carbon Dots from Tartaric Acid and Various Alkylol Amines under Microwave Irradiation,” Nanoscale 7 (2015): 15915–15923. 26364977 10.1039/c5nr04209g

[57] Y. Jiang , Y. Wang , F. Meng , B. Wang , Y. Cheng , and C. Zhu , “N‐Doped Carbon Dots Synthesized by Rapid Microwave Irradiation as Highly Fluorescent Probes for Pb^2+^ Detection,” New Journal of Chemistry 39 (2015): 3357–3360.

[58] S. Sajjadi , A. Khataee , R. D. C. Soltani , and A. Hasanzadeh , “N, S Co‐Doped Graphene Quantum Dot–decorated Fe3O4 Nanostructures: Preparation, Characterization and Catalytic Activity,” Journal of Physics and Chemistry of Solids 127 (2019): 140–150.

[59] A. Jiananda , E. K. Sari , D. A. Larasati , et al., “Optical, Microstructural, and Magnetic Hyperthermia Properties of Green‐Synthesized Fe3O4/Carbon Dots Nanocomposites Utilizing Moringa Oleifera Extract and Watermelon Rinds,” Carbon Trends 13 (2023): 100305.

[60] R. Fattahi Nafchi , R. Ahmadi , M. Heydari , M. R. Rahimipour , M. J. Molaei , and L. Unsworth , “In Vitro Study: Synthesis and Evaluation of Fe3O4/CQD Magnetic/Fluorescent Nanocomposites for Targeted Drug Delivery, MRI, and Cancer Cell Labeling Applications,” Langmuir 38, no. 12 (2022): 3804–3816. 35294836 10.1021/acs.langmuir.1c03458

[61] S. Ahmadian‐Fard‐Fini , M. Salavati‐Niasari , and D. Ghanbari , “Hydrothermal Green Synthesis of Magnetic Fe3O4‐Carbon Dots by Lemon and Grape Fruit Extracts and as a Photoluminescence Sensor for Detecting of E. Coli Bacteria,” Spectrochimica Acta Part A: Molecular and Biomolecular Spectroscopy 203 (2018): 481–493. 29898431 10.1016/j.saa.2018.06.021

[62] A. I. Pavón‐Hernández , E. Rodríguez‐Velázquez , M. Alatorre-Meda , et al., “Magnetic Nanocomposite with Fluorescence Enhancement Effect Based on Amino Acid Coated‐Fe3O4 Functionalized with Quantum Dots,” Materials Chemistry and Physics 251 (2020): 123082.

[63] S. Koley , P. K. Risla Sherin , M. Nayak , N. Barooah , A. C. Bhasikuttan , and J. Mohanty , “Arene‐Functionalized Gold Nanoparticles: Applications in Drug Delivery and Bioimaging,” ACS Physical Chemistry Au 4, no. 5 (2024): 522–530. 39364352 10.1021/acsphyschemau.4c00027 PMC11447960

[64] R. Atchudan , T. N. J. I. Edison , K. R. Aseer , S. Perumal , N. Karthik , and Y. R. Lee , “Highly Fluorescent Nitrogen‐Doped Carbon Dots Derived from Phyllanthus Acidus Utilized as a Fluorescent Probe for Label‐Free Selective Detection of Fe3+ Ions, Live Cell Imaging and Fluorescent Ink,” Biosensors and Bioelectronics 99 (2018): 303–311. 28780346 10.1016/j.bios.2017.07.076

[65] L. Wang , Y. Yin , A. Jain , and H. S. Zhou , “Aqueous Phase Synthesis of Highly Luminescent, Nitrogen‐Doped Carbon Dots and Their Application as Bioimaging Agents,” Langmuir 30 (2014): 14270–14275. 25365539 10.1021/la5031813

