# Supplementary material for: Luminescent Fe3O4 Nanohybrid for Intra‐Cellular Imaging and Combinatorial Chemo–Photothermal Therapy in Cancer
Source: Chembiochem. 2026 Apr 21;27(8):e202500886. doi: 10.1002/cbic.202500886 (PMC13096865; doi:10.1002/cbic.202500886)
Supplement: Supplementary file 1 — Supplementary Material [file CBIC-27-e202500886-s001.pdf]

Supporting information

The authors have cited additional references within the Supporting Information. [64,65]

Luminiscent Fe_3O_4 Nanohybrid for Intra-Cellular Imaging and Combinatorial Chemo-Photothermal Therapy in Cancer

Bijaideep Dutta*[a,b], Sonali Gupta [a,b], Sharanaya Purandare [a], Premlata Bind [a], Rudheer Bapat[c], N. K. Prasad[d], K. C. Barick[a,b]*, P. A. Hassan

[a]Chemistry Division, Bhabha Atomic Research Centre, Trombay, Mumbai-400085, India

[b]Homi Bhabha National Institute, Anushaktinagar, Mumbai - 400094, India

[c]Dept. of Condensed Matter Physics and Materials Science, Tata Institute of Fundamental Research, Mumbai, India

[d]Department of Metallurgical Engineering, IIT BHU, Varanasi, India

**E-mail: bijaideep@barc.gov.in,kcbarick@barc.gov.in Tel.: + 91 22 2559 1922*

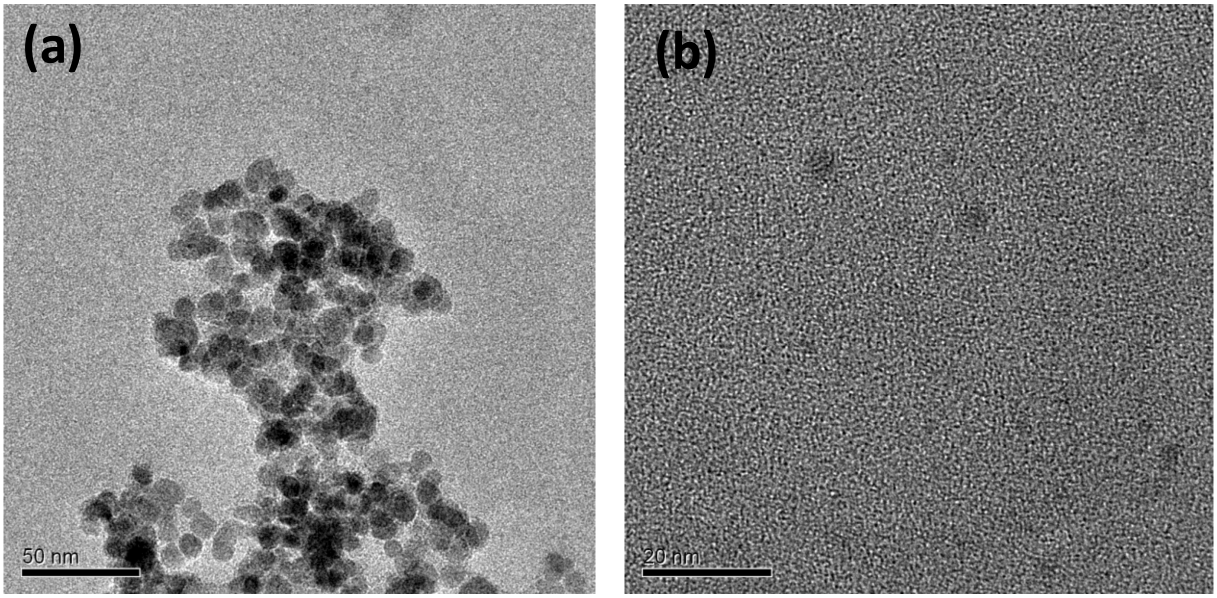

Supporting info S1: *TEM images of (a) bare Fe_3O_4 and (b) GUCDs nanoparticles*

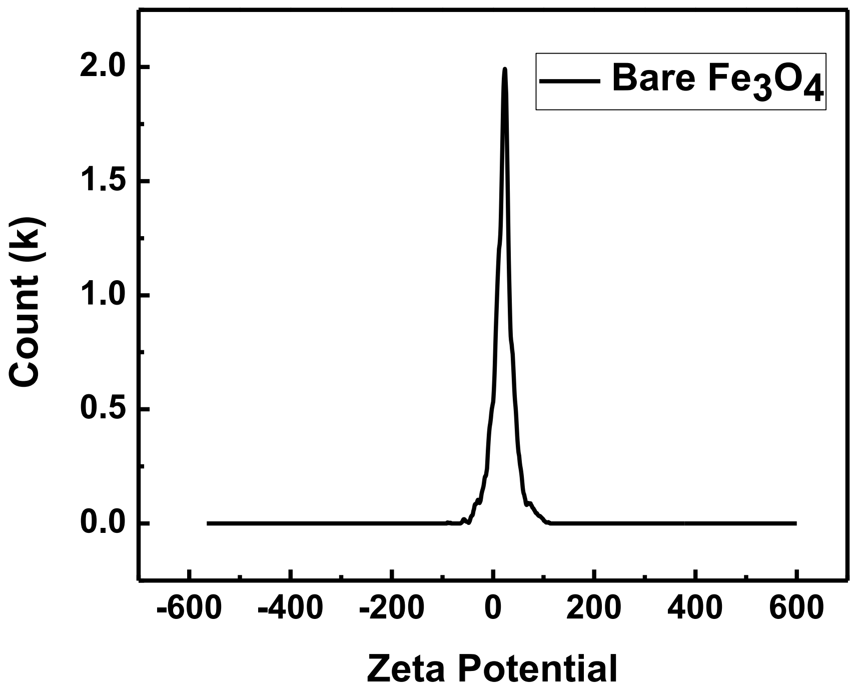

Supporting info S2: *zeta potential of bare Fe_3O_4 nanoparticle in nanopure water medium*

pH	***Zeta potential (mV)***	
	GUCDs	***FCDs***
2	*3.2*	*26.1*
4	*- 0.6*	*22.4*
6	*- 11.9*	*- 8.9*
8	*- 20.1*	*- 11.4*
10	*- 30.2*	*- 31.5*
H_2O	*-18.4*	*-7.6*

Supporting info ST1: *zeta potential of GUCDs and FCDs at various pH medium*

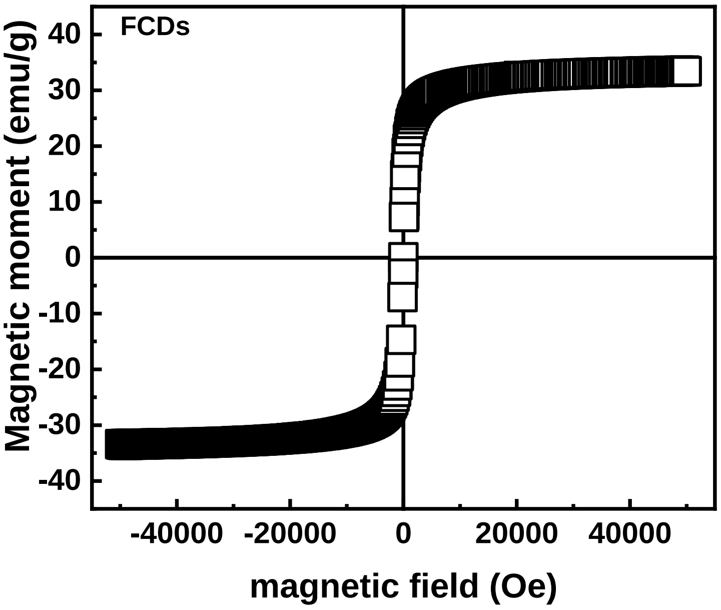

Supporting info S3: *magnetic measurement data of FCDs NPs*

Measurements of fluorescence quantum yield

The QY of the synthesized FNCDs was measured based on an established procedure [64,65]. In brief, quinine sulfate in 0.1 M H_2SO_4 was used as the reference standard. The QY was calculated according to the following equation (1):

$$QY\,(\%) = QY_R\ \frac{I_S A_R (n_S)^2}{I_R A_S (n_R)^2} \qquad (1)$$

where, “I” is the measured integrated fluorescent emission intensity, “n” is the refractive index of the solvent, and “A” is the absorbance. The subscript “R” and “S” refers to the corresponding parameter of known fluorescent standard and for the synthesized sample, respectively.

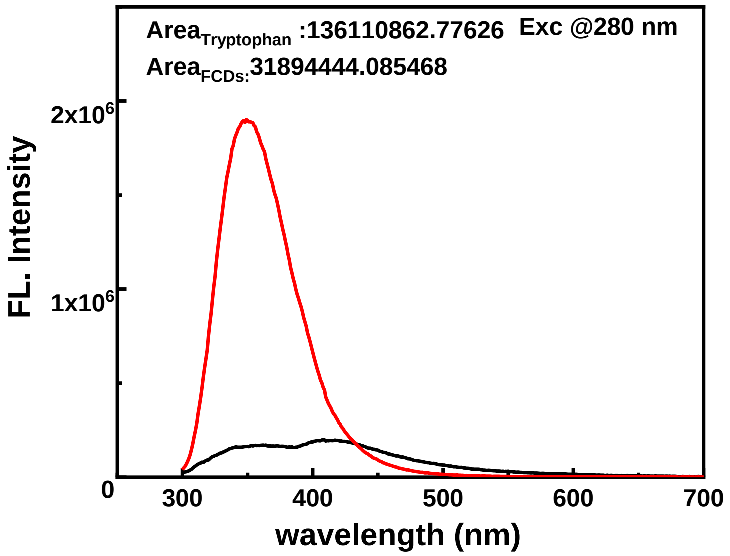

Supporting info S4: *Fl. Quantum yield of FCDs*

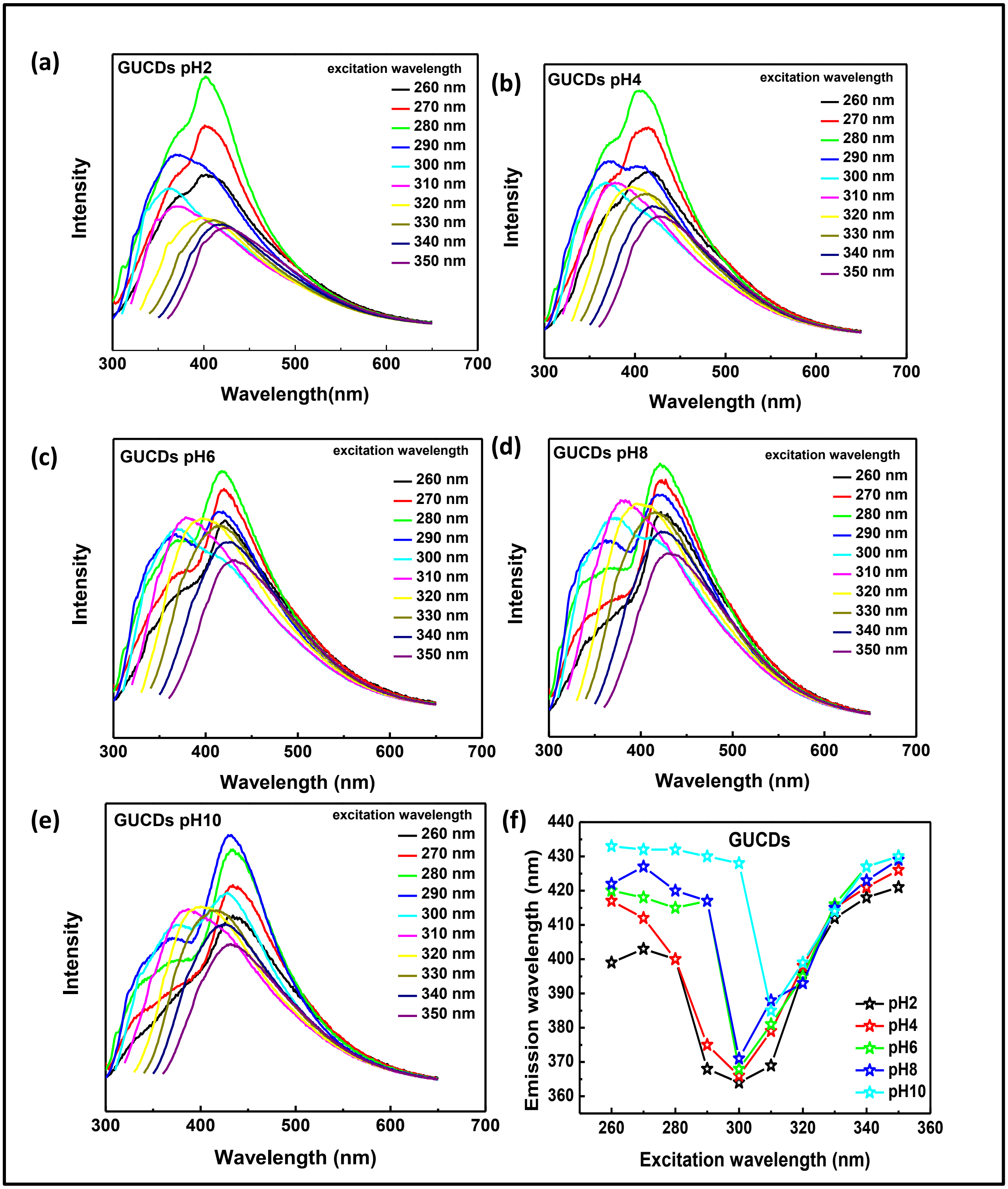

Supporting info S5: *(a-e) Excitation dependent fluorescence emission spectra of GUCDs at different pH and (f) their excitation vs. emission wavelength graph*

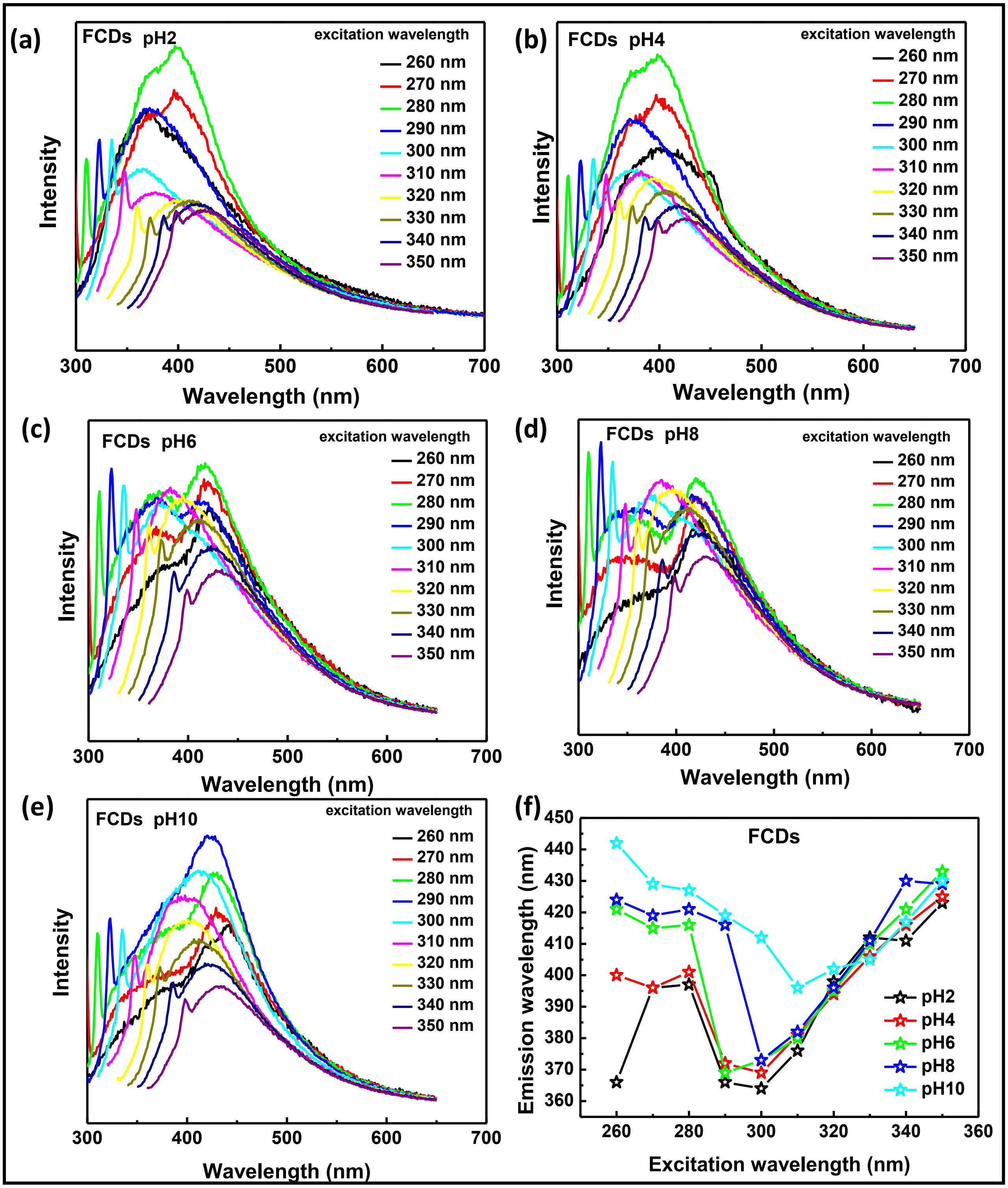

Supporting info S6. (a-e) *Excitation dependent fluorescence emission spectra of FCDs at five different pHs (2-10) and (f) their excitation vs. emission wavelength graph*

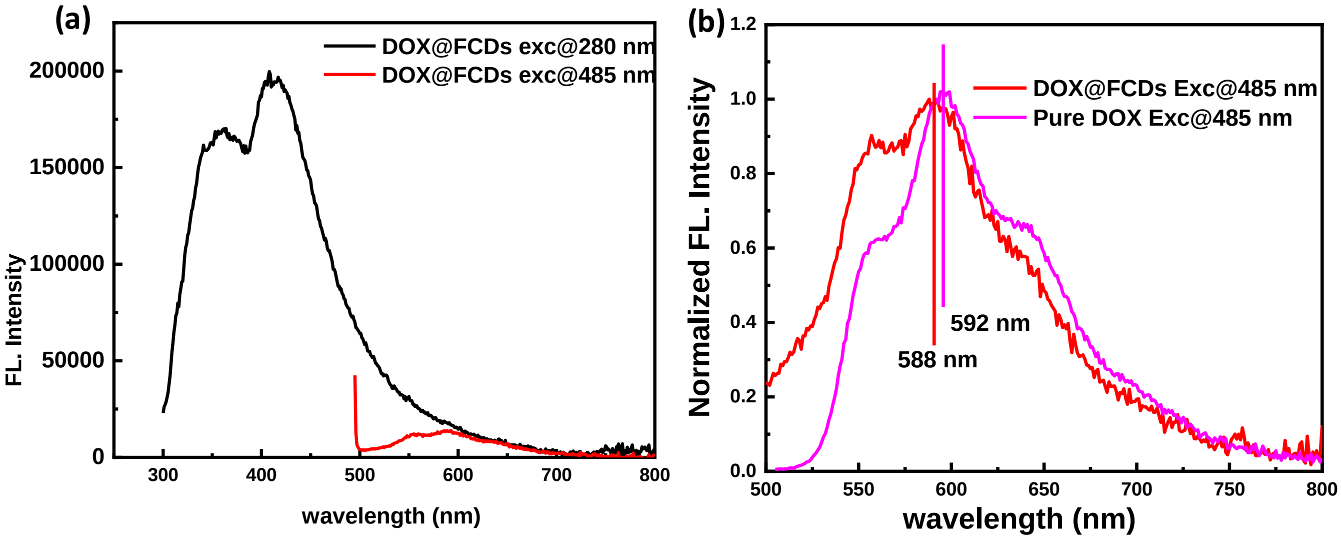

Supporting info S7. (a) Emission spectra of DOX@FCDs at 280 nm and 485 nm respectively, (b) Comparison of emission maxima of pure DOX and DOX@FCDs upon excitation with 485 nm.

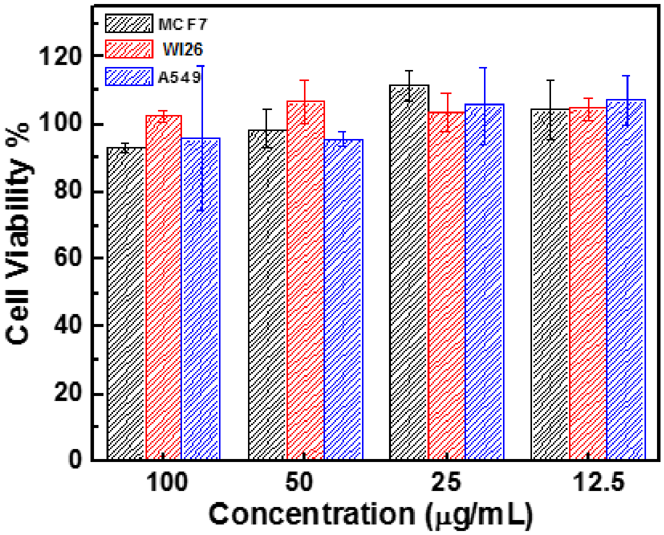

Supporting info S8. *Biocompatibility study via MTT of synthesized FCDs NPs. Data represent the mean ±SD (n= 3).*

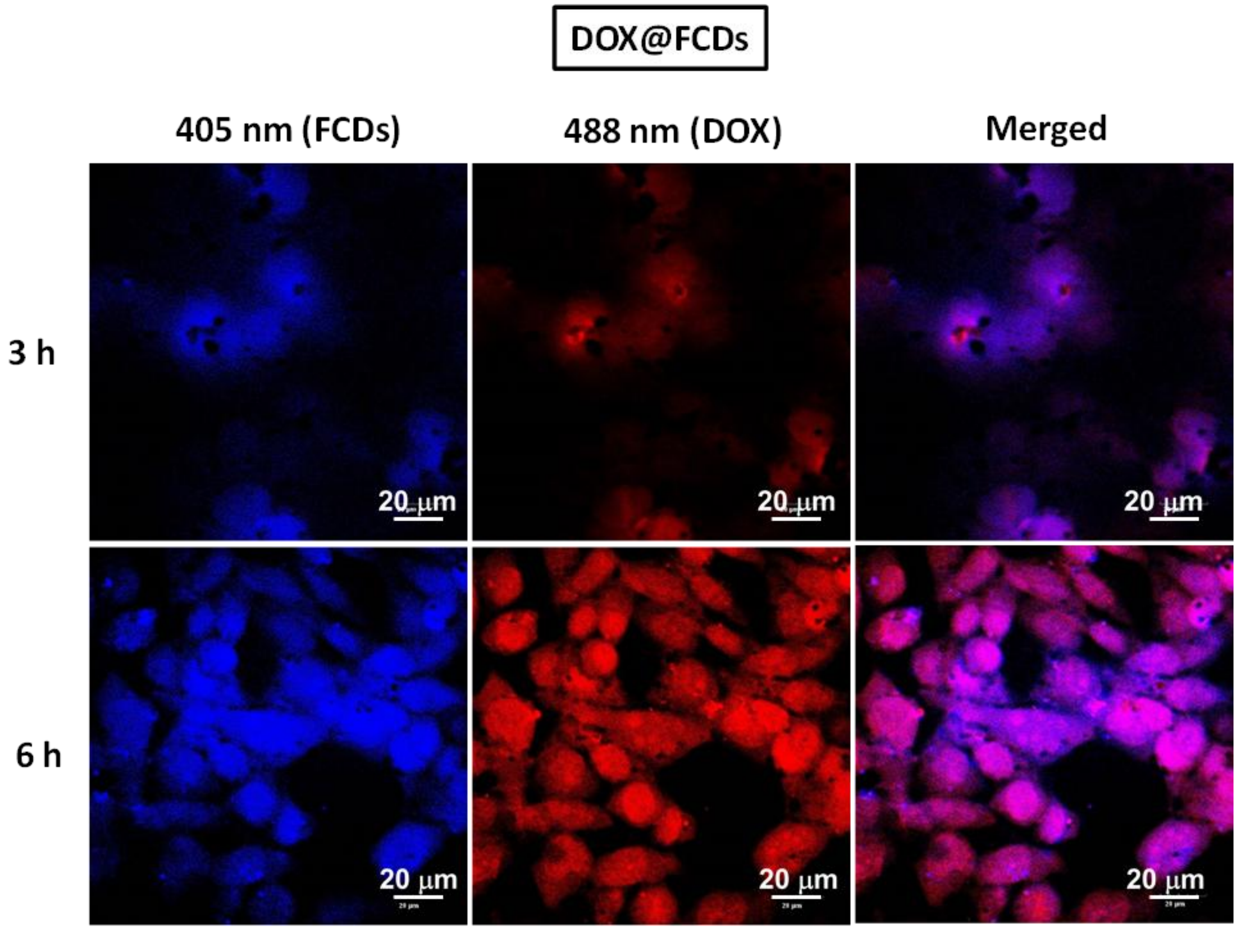

Supporting info S9. *Confocal microscopy images of MCF-7 cells after incubation with DOX@FCD NPs under culture conditions at two different time frame (a) 3 and (b) 6 h)(red filter for DOX and blue filter for FCDs).*
